# Evaluation of Steroid Pulse Therapy Responsiveness in Myelin-Oligodendrocyte Glycoprotein Antibody-Positive Optic Neuritis

**DOI:** 10.7759/cureus.56673

**Published:** 2024-03-21

**Authors:** Yasuyuki Takai, Akiko Yamagami, Mayumi Iwasa, Kenji Inoue, Masato Wakakura, Toshiyuki Takahashi, Keiko Tanaka

**Affiliations:** 1 Department of Ophthalmology, Inouye Eye Hospital, Tokyo, JPN; 2 Department of Neurology, Tohoku University Graduate School of Medicine, Sendai, JPN; 3 Department of Animal Model Development, Brain Research Institute, Niigata University, Niigata, JPN

**Keywords:** visual field defect, steroid pulse therapy, steroid, myelin-oligodendrocyte glycoprotein antibody, optic neuritis

## Abstract

Purpose: Myelin-oligodendrocyte glycoprotein antibody-positive optic neuritis (MOGON) is usually responsive to the steroid, but, for some patients, steroid pulse therapy alone may be inadequate. This study aimed to investigate the factors predicting the response to steroid pulse therapy in MOGON.

Methods: This study included 17 patients (24 eyes) with MOGON, who received single steroid pulse therapy as initial treatment. Best corrected visual acuity (BCVA) and mean deviation (MD) values after treatment were examined concerning findings at onset.

Results: No correlation was found between BCVA at onset and after treatment, but a correlation was observed between MD values at onset and after treatment (correlation coefficient 0.48, p=0.01, Spearman's rank correlation coefficient). Age, gender, duration from onset to treatment, magnetic resonance imaging findings, and optical coherence tomography findings did not affect visual function after treatment.

Conclusions: Severe visual field impairment at onset may indicate that additional treatment may be necessary.

## Introduction

Myelin-oligodendrocyte glycoprotein antibody-positive optic neuritis (MOGON) causes inflammatory demyelination of the optic nerve, leading to visual dysfunction. Clinically, it often presents with papilledema and ocular pain, and on MRI, it is associated with perineuritis and extensive lesions [1]. Steroid pulse therapy is commonly used in acute-phase treatment. While the response to steroids is usually positive, some cases respond inadequately and may require additional steroid pulse therapy, high-dose intravenous immunoglobulin, or plasma exchange therapy [2]. When MOGON is suspected, antibody testing is considered, but it takes time to obtain results, necessitating a relatively early decision on additional treatment after an initial course of steroid pulse therapy. However, currently, there are no clear factors to predict the response to steroid pulse therapy at the onset of MOGON. This study investigated factors related to the responsiveness to steroid pulse therapy based on the findings at the onset of MOGON.

## Materials and methods

Study design and patients

From the cases of optic neuritis that visited Inouye Eye Hospital, 24 patients were identified as myelin-oligodendrocyte glycoprotein (MOG) antibody-positive and had undergone steroid pulse therapy (methylprednisolone 1000 mg for three days) as acute phase treatment. Among these patients, the study focused on 17 patients with 24 eyes (limited to the initially affected eyes) who had visual acuity tests and Humphrey visual field tests (30-2 program) conducted both before and after treatment and who did not have other retinal or optic nerve diseases. Inouye Eye Hospital’s institutional review board issued approval 202401-1.

Clinical assessment

Optic neuritis was diagnosed based on the interviews, medical history, and ophthalmic examinations (visual acuity, intraocular pressure, flicker fusion threshold, state of relative afferent pupillary defect, slit-lamp examination, fundus examination, visual field test, fundus fluorescein angiography, orbital MRI) following the report by Petzold et al. [3]. MOG antibody measurement was performed after obtaining informed consent, with blood samples taken before treatment for optic neuritis. The samples were kept on ice after collection and transported to the Department of Neurology at Niigata University Medical Faculty or Tohoku University Medical Faculty, where MOG antibody tests were conducted [4,5]. MOG antibody positivity was determined qualitatively using a cell-based assay (CBA) and included cases that met the criteria reported by Banwell et al. [6]. 

**Figure 1:**
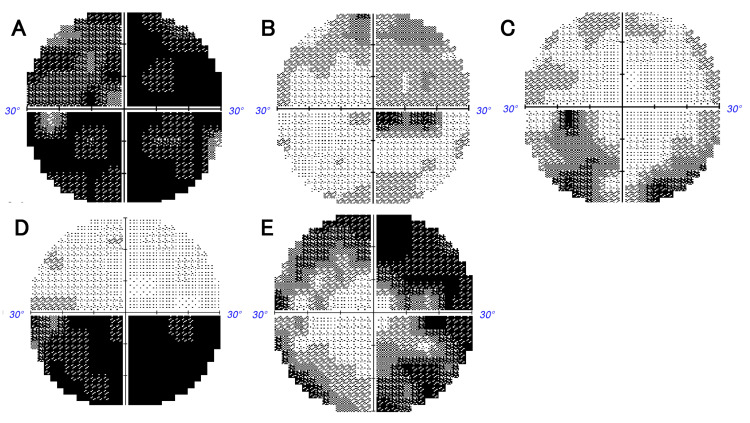
Classifications for optic nerve visual field abnormalities. (A) Generalized depression (or severe central scotoma), (B) centrocecal scotoma, (C) retinal nerve fiber layer defects, (D) hemianopic, and (E) peripheral constriction.

Best corrected visual acuity (BCVA) was represented in logMAR (logarithm of the minimum angle of resolution) units, with counting fingers equated to 2.6 logMAR, hand motion to 2.9 logMAR, light perception to 3.1 logMAR, and no light perception to 3.4 logMAR [7]. Visual field tests were conducted using the Humphrey Field Analyzer (30-2 program), and qualitative assessments of visual field defects, as well as mean deviation (MD) values, were measured. The qualitative patterns of visual field defects in acute optic neuritis were assessed in Figure 1 based on the previous reports [8,9].

Optical coherence tomography (OCT) was performed to capture disc images, and the circumpapillary retinal nerve fiber layer (cpRNFL) was categorized and measured in superior, temporal, inferior, and nasal quadrants, evaluating the average thickness of cpRNFL.

**Figure 2:**
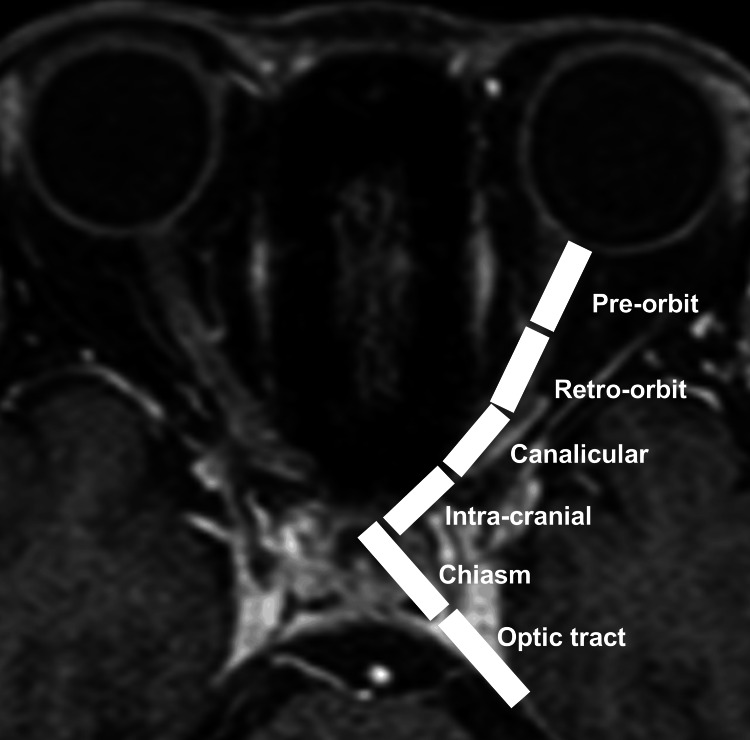
Definition of the MRI lesion segments. The optic nerve was divided from anterior to posterior by pre-orbit, retro-orbit, canalicular, intra-cranial, chiasm, and optic tract.

In orbital MRI, the optic nerve is typically divided into the following segments: pre-orbit (anterior within the orbit), retro-orbit (posterior within the orbit), canalicular (within the optic canal), intra-cranial (within the cranium), chiasm (optic chiasm), and optic tract [10]. Imaging was performed to observe these details, and the presence or absence of lesions in each segment was assessed, and the approximate length of optic nerve lesions was scored from zero to six segments (Figure 2). Eyes with lesions in two or fewer segments were defined as the short lesion group, while those with lesions in three or more segments were defined as the long lesion group.

In MOGON, we measured visual function (BCVA and MD values) following steroid pulse therapy and examined factors present at disease onset that could be involved in post-treatment visual dysfunction. The timing for assessing visual function after steroid pulse therapy was defined as one to two weeks post-treatment, referencing the clinical trial protocols for high-dose intravenous immunoglobulin therapy [11].

Statistical analysis

Statistical analyses were conducted using the software Statistical Package for Social Sciences (SPSS), version 22.0 (IBM Corp., Armonk, NY). Data were reported as medians and ranges and were treated as nonparametric data. The criterion for statistical significance was a p-value below 0.05.

## Results

Clinical findings

Table 1 summarizes the clinical findings. The age of the patients was 42 (range 22-70) years, with six males (nine eyes) and 11 females (15 eyes). The duration from onset to treatment was five (range 1-18) days. OCT was available for 18 eyes, with an average cpRNFL thickness of 116 (range 52.25-288) μm. Orbital MRI was available for 20 eyes, and lesion length was 3.5 (range 1-5) segments; there were eight eyes in the short lesion group and 12 eyes in the long lesion group.

**Table 1:** Clinical characters at onset. cpRNFL: Circumpapillary retinal nerve fiber layer, BCVA: Best corrected visual acuity,  logMAR: Logarithm of the minimum angle of resolution, MD: Mean deviation.

Number of the cases	Seventeen cases (24 eyes)
Age at onset	42 (22, 70) years old
Gender	6:11 (male:female)
Duration from onset to treatment	5 (1,18) days
cpRNFL	116（52.25,288）μm
MRI	3.5（1, 5）segment
Short lesions	8 eyes
Long lesions	12 eyes
BCVA	0.52 (-0.08, 2.8) logMAR
MD values	-19.05 (-31.9, -2.26) dB

Visual function after treatment

**Figure 3:**
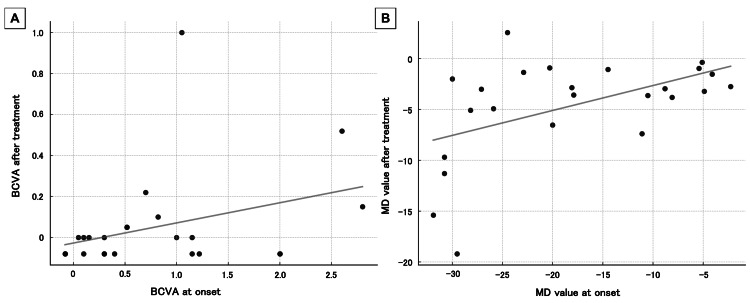
Correlation of visual function before and after treatment A: The visual acuity at onset was 0.52 (range -0.08 to 2.8) logarithm of the minimum angle of resolution (logMAR), and after treatment, it was 0 (range -0.08 to 1) logMAR. No correlation was found between visual acuity at onset and post-treatment (correlation coefficient 0.34, p=0.11). B: The MD value at onset was -19.05 (range -31.9 to -2.26) dB, and -3.12 (range -19.2 to 2.56) dB at post-treatment. The correlation was observed between the MD values at onset and post-treatment (correlation coefficient 0.48, p=0.01). Spearman's rank correlation coefficient, significance level p<0.05. BCVA: Best corrected visual acuity, logMAR: Logarithm of the minimum angle of resolution, MD: Mean deviation.

At the initial consultation, the visual acuity was 0.52 (range -0.08 to 2.8) logMAR, and the MD value was -19.05 (range -31.9 to -2.26) dB. Post-treatment visual acuity improved to 0 (range -0.08 to 1) logMAR, and MD value improved to -3.12 (range -19.2 to 2.56) dB, showing improvement compared with the initial visual acuity and MD values (p<0.01, Wilcoxon signed-rank test). No correlation was observed between initial and post-treatment visual acuity (correlation coefficient 0.34, p=0.11, Spearman's rank correlation coefficient: Figure 3A). A statistically significant correlation was found between initial and post-treatment MD values (correlation coefficient 0.48, p=0.01, Spearman's rank correlation coefficient: Figure 3B). 

At the initial consultation, the pattern of visual field defect was retinal nerve fiber layer defect in 10 eyes, and generalized depression (or severe central scotomas) was in nine eyes. After treatment, visual field defects resolved in 14 eyes, with the remaining defects most commonly retinal nerve fiber layer defects in six eyes (Table 2).

**Table 2:** Visual defect patterns before and after treatment

Before treatment		After treatment	
Generalized depression	9	Normal	14
Centrocecal scotoma	3	Centrocecal scotoma	2
Retinal nerve fiber layer defects	10	Retinal nerve fiber layer defects	6
Peripheral constriction	1	Peripheral constriction	2
Hemianopic	1		

We examined whether age at onset, gender, and the duration before treatment affected post-treatment visual function. No correlation was observed between age and post-treatment BCVA or MD values (BCVA, correlation coefficient -0.195, p=0.36; MD value, correlation coefficient -0.19, p=0.37, Spearman's rank correlation coefficient). There was no significant difference in post-treatment visual function between genders (BCVA, male vs. female; 0 (range -0.08 to 0.52) logMAR vs. -0.08 (range -0.08 to 1) logMAR, p=0.24; MD value, male vs. female, -3.63 (range -19.2 to 2.56) dB vs. -2.95 (range -15.4 to -0.35) dB, p=0.35, Mann-Whitney U test). There was no significant correlation between the duration before treatment and post-treatment visual function (BCVA, correlation coefficient -0.12, p=0.57; MD value, correlation coefficient -0.18, p=0.39, Spearman's rank correlation coefficient).

We investigated how the thickness of the average cpRNFL measured by OCT before treatment affects the recovery of visual function after treatment. No significant correlation was found between the cpRNFL thickness and post-treatment visual function (BCVA, correlation coefficient -0.195, p=0.36; MD value, correlation coefficient -0.19, p=0.37, Spearman's rank correlation coefficient). We also investigated how the length of lesions observed on MRI before treatment affects the recovery of visual function after treatment. There was also no difference between the short and long lesion groups in MRI lesion length (BCVA, short lesion group vs. long lesion group, -0.04 (range -0.08 to 0.22) logMAR vs. 0 (range -0.08 to 1) logMAR, p=0.55; MD value, short lesion group vs. long lesion group, -2.85 (range -5.1 to -0.35) dB vs. -3.72 (range -19.2 to 2.56) dB, p=0.16, Mann-Whitney U test).

## Discussion

In this study, we used the BCVA and MD values one to two weeks after a single course of steroid pulse therapy as indicators of treatment responsiveness. The findings demonstrated that lower mean deviation values at onset correlated with less favorable MD values following steroid pulse therapy. No significant differences were observed when considering age, gender, the duration before treatment, MRI lesion length, or cpRNFL thickness at onset. It is necessary to consider the additional treatment after a single course of steroid pulse therapy, especially in cases with severe visual field impairment at onset.

Although visual function with optic neuritis often improves with treatment, a 15-year follow-up study of optic neuritis has reported cases with residual visual field defects [9]. Similarly, in MOGON, it has been reported that while visual prognosis is generally good, some cases may have persistent visual field abnormalities [12]. While visual acuity is often the primary outcome measure in evaluating the effectiveness of treatment for optic neuritis, including MOGON, the persistence of visual symptoms due to residual visual field defects should be recognized. Our study found a tendency for poorer post-treatment MD values with worse initial MD values, highlighting the need for careful monitoring of visual field improvements throughout the treatment process.

In our study, no central scotoma was observed after steroid pulse therapy. Optic neuritis often results in central scotoma, but in MOGON, central scotoma quickly resolves after steroid pulse therapy [8]. MOGON tends to show long lesions and optic perineuritis as seen in MRI studies, and severe optic disc edema as seen with OCT [13]. It showed severe inflammation of the optic nerve at onset. Pathological studies of myelin oligodendrocyte glycoprotein antibody-associated disease (MOGAD) have reported that MOG was expressed on the outermost layer of the myelin sheath of myelin where MOG resides, MOG antibody-associated demyelination may initially occur on the surface of the myelin sheath [14]. The relative preservation of oligodendrocytes was also confirmed in demyelinating lesions. The prompt regression of inflammation in response to anti-inflammatory treatments may indicate that the myelin damage may be minor despite the severe inflammation at the onset, potentially explaining the observed resolution of the central scotoma. As in our current study, the severe visual field impairment at onset may lead to persistent visual field impairments even after steroid pulse therapy, so the severity of visual field impairment at onset may serve as a useful indicator of myelin damage, rather than the length of the lesion on MRI or the degree of optic disc edema observed with OCT.　　

In MOGON, it is common to administer a single course of steroid pulse therapy during the acute phase. If the response to treatment is poor, additional steroid pulse therapy or an extension to five days is often considered. For refractory cases, other options such as high-dose intravenous immunoglobulin therapy or plasma exchange therapy are considered. The efficacy of intravenous immunoglobulin therapy for optic neuritis has been reported [11]. Similarly, good responsiveness to intravenous immunoglobulin therapy in MOGON has been documented, making it a potential option for additional treatment in refractory cases [15]. Considering this report, it is essential to keep in mind the possibility of additional treatment for cases with poor initial MD values.

This study has several limitations. Due to its retrospective design and small sample size, complete OCT and MRI data could not be obtained for all patients. Also, cases with severe visual function impairment at onset often underwent Goldman visual field testing, which could have introduced a bias in our study when only considering Humphrey visual field test results, potentially leading to a decrease in the number of cases. There are reports suggesting that a shorter duration from the onset of vision loss to treatment correlates with better post-treatment visual function, but, by limiting to the initial attack only, we may have arrived at different results compared to previous reports [2]. Chen et al. also mentioned that when post-treatment visual acuity was adjusted for visual acuity at the time of an attack, no difference was found due to the duration from the attack to treatment, and it has been suggested that worse visual function at the time of an attack can lead to worse post-treatment visual function [2]. To reach higher levels of evidence-based conclusions, future prospective randomized controlled trials with a larger number of patients will be necessary.

## Conclusions

In cases of myelin-oligodendrocyte glycoprotein antibody-positive optic neuritis (MOGON) with severe visual field impairment at onset, the visual field deficits may remain following steroid pulse therapy. In such cases, additional treatment should be considered. To clarify the steroid responsiveness of MOGON, further accumulation of cases is desired.
